# Durability Analysis of Cold Spray Repairs: Phase I—Effect of Surface Grit Blasting

**DOI:** 10.3390/ma17112656

**Published:** 2024-05-31

**Authors:** Daren Peng, Caixian Tang, Jarrod Watts, Andrew Ang, R. K. Singh Raman, Michael Nicholas, Nam Phan, Rhys Jones

**Affiliations:** 1 ARC Industrial Transformation Training Centre on Surface Engineering for Advanced Materials, Faculty of Science, Engineering and Technology, Swinburne University of Technology, John Street, Hawthorn, VIC 3122, Australiaaang@swin.edu.au (A.A.); 2 Centre of Expertise in Structural Mechanics, Department of Mechanical and Aeronautical Engineering, Monash University, Wellington Rd, Clayton, VIC 3800, Australia; 3 Rosebank Engineering Australia, 836 Mountain Highway, Bayswater, VIC 3153, Australia; 4 US Army Research Laboratory, U.S. Army Combat Capabilities Development Command Weapons and Materials Research Directorate, Aberdeen Proving Ground, Aberdeen, MD 20852, USA; 5 Structures Division, Naval Air Systems Command, Patuxent River, MD 20670, USA

**Keywords:** cold spray, skin corrosion, durability, MIL-STD-1530Dc, EZ-SB-19-01, JSSG2006

## Abstract

This paper presents the results of an extensive investigation into the durability of cold spray repairs to corrosion damage in AA7075-T7351 aluminium alloy specimens where, prior to powder deposition, the surface preparation involved grit blasting. In this context, it is shown that the growth of small naturally occurring cracks in cold spray repairs to simulated corrosion damage can be accurately computed using the Hartman–Schijve crack growth equation in a fashion that is consistent with the requirements delineated in USAF Structures Bulletin EZ-SB-19-01, MIL-STD-1530D, and the US Joint Services Structural Guidelines JSSG2006. The relatively large variation in the *da*/*dN* versus Δ*K* curves associated with low values of *da*/*dN* highlights the fact that, before any durability assessment of a cold spray repair to an operational airframe is attempted, it is first necessary to perform a sufficient number of tests so that the worst-case small crack growth curve needed to perform the mandated airworthiness certification analysis can be determined.

## 1. Introduction

Cold spray, also known as supersonic particle deposition (SPD), is increasingly being used to repair both rotary and fixed-wing aircraft [1,2,3,4,5,6,7,8,9,10]. However, as delineated in the United States Joint Services Structural Guidelines JSSG2006 [11], MIL-STD-1530D [12], and USAF Structures Bulletin EZ-SB-19-01 [13], the airworthiness certification of a cold spray repair requires a durability assessment. Furthermore, as per MIL-STD-1530D and USAF Structures Bulletin EZ-SB-19-01, the durability and damage tolerance assessment should be based on linear elastic fracture mechanics (LEFM). Here, the term ‘durability’ is taken to be as defined in MIL-STD-1530D and JSSG2006: “Durability is the attribute of an airframe that permits it to resist cracking for a prescribed period of time”.

As such, the purpose of this paper is to illustrate how to perform an LEFM-based durability assessment of a cold spray repair using a “building block” approach that is consistent with that mandated in JSSG2006 and MIL-STD-1530D. In this context, it should be noted that [14,15,16,17] revealed that cold spray coatings are exceptionally damage tolerant and that failure often occurs due to the nucleation, and subsequent growth, of cracking at the intersection between the substrate being repaired and the cold spray repair. Furthermore, the damage tolerance of cold spray repairs to AA7075-T7351 substrates is such that even when the cold spray coating was notched, the failure was due to the nucleation and subsequent growth of cracks in the substrate, and that the cold spray deposition did not crack or delaminate until close to the final failure of the specimens [17].

As noted in MIL-STD-1530D and EZ-SB-19-01, analysis is the key to certification, and the role of testing is merely to validate or correct the analysis. However, there are only a few papers [10,16] that present a linear elastic fracture mechanics (LEM)-based durability analysis of a cold spray repair, where the initial crack length is of the order of the equivalent initial damage size (EIDS) that is mandated, namely 0.0254 mm (0.01 inch) [11,12,13]. Similarly, other than [10,16], there are no papers in which the predicted crack growth histories are compared with experimental measurements. However, in [10], the nucleating cracks are associated with corrosion pitting down the bore of a fastener hole that contains (existing) intergranular corrosion. As such, these cracks are not initiated either in the cold spray repair, or at the intersection between the cold spray and the substrate being repaired. Consequently, aside from the preliminary study [16] that presented an initial study of a small number of cold-spray-repaired specimens, there are currently no published papers presenting a durability analysis of cold spray repairs where failure was due to the nucleation and subsequent growth of cracks at the intersection between the cold spray coating and the substrate. There also no publications in which

(i) the analysis was performed using LEFM;
(ii) the initial crack sizes were smaller than or comparable to the mandated EIDS;
(iii) the experimentally measured crack growth histories were compared with predictions;
(iv) and the experimental test results were used to generate the variability in the *da*/*dN* versus Δ*K* curves that are needed to enable a worst-case analysis, as mandated in the NASA Fracture Control Handbook NASA-HDBK-1510 [18].

Consequently, the purpose of this paper is to illustrate how to perform the necessary durability analysis in a fashion that is consistent with USAF Structures Bulletin EZ-SB-19-01, MIL-STD-1530D, and Joint Services Structural Guidelines JSSG2006. 

To achieve this objective, this paper presents the results of an extensive test program addressing cold spray repairs to simulated corrosion damage. To this end, tests on twelve cold spray repairs to AA7075-T7351 specimens containing a simulated width corrosion cut were performed. To enable the crack growth histories to be determined, the specimens were fatigue tested using six different marker block load spectra. This resulted in the nucleation and subsequent growth of twenty-five (25) fatigue cracks, which, as previously observed [14,15,16,17], nucleated in the AA7075-T7351 substrate at the interface between the substrate and the cold spray deposit.

## 2. Materials and Methods

### 2.1. The Geometric Dimensions and the Applied Marker Block Load Spectra

Tests on twelve cold spray repairs to AA7075-T7351 specimens containing a simulated width corrosion cut were performed. These specimens were labelled as follows: 75_1_NC_1_#2, 75_1_NC_1_#3, 75_1_NC_1_#4, 75_1_NC_1_#5, 75_1_NC_2_#3, 75_1_NC_2_#4, 75_1_NC_2_#5, B_1_1_#1, B_1_1_#2 and B_1_1_#3; see Table 1. The dimensions and geometry of the test specimens used in this study are shown in Figure 1.

The cold spray deposition was performed using the VRC Metal System Brolga mobile cold spray system [19]. This system was chosen as it is in use at the US Navy Fleet Readiness Center Southwest (FRCSW) [20]. The aluminium alloy 7075 had a particle size range of 15–53 µm. The cold spray parameters were optimized to produce a porosity level below 0.5% and a minimum adhesion strength of 26 MPa on a AA7075-T7351 substrate (The precise details associated with the deposit process are commercial in confidence). The coating hardness was measured as 82–84 HRB.

To establish the crack growth histories, the specimens were fatigue tested using six different marker block load spectra, as shown in Table 1 and Table 2. For all specimens, the nucleation and subsequent growth of the twenty-five fatigue cracks were found to be at the interface between the AA7075-T7351 substrate and the AA7075 cold spray deposit, and were consistent with previous experiments [14,15,16,17].

**Table 1:** Block loading spectrum.

Spectrum	P_max_	Cycles		Repeat Blocks	Cycles		Repeat Blocks	Cycles		Repeat Blocks
	kN	R = 0.1	R = 0.8		R = 0.2	R = 0.8		R = 0.2	R = 0.8	
1	35	5000	18,000	To failure	-	-	-	-	-	-
2	30	500	18,000	To failure	-	-	-	-	-	-
3 *	30	300	15,000	To failure	-	-	-	-	-	-
4	26.77	500	18,000	To failure	-	-	-	-	-	-
5	26.77	300	15,000	To failure	-	-	-	-	-	-
6	26.77	300	15,000	15	200	10,000	15	100	3000	To failure

* A schematic diagram of these particular test spectra is given in Figure 2.

### 2.2. Stress Analysis and Durability Analyses

As in previous works [10,16] that also studied the growth of cracks in cold spray repairs to AA7075-T7351 aluminium alloy components, the analysis used the Hartman–Schijve crack growth equation [21] to compute the crack growth histories for each of the twenty-five cracks that nucleated and grew in these tests. This equation takes the following form:

*da*/*dN* = *D* (∆*K* − ∆*K_thr_*)/√(1 − *K_max_*/*A*))*^n^* (1)

Here, ∆*K* = *K_max_* – *K_min_*, where *K_max_* and *K_min_* are the maximum and minimum values of the stress intensity factor (*K*) in a cycle, ∆*K_thr_* is the fatigue threshold, *n* and *D* are the material constants, and *A* is the apparent cyclic fracture toughness; see [21] for more details. The application of this formulation to a wide range of problems can be found in other journal papers [22,23,24,25,26,27,28,29,30,31,32,33,34,35,36,37,38,39,40]. The relationship between this formulation and crack-closure-based crack growth equations [41,42,43,44,45,46,47,48,49,50,51,52,53,54,55] is presented in [55]. A number of crack growth equations that are similar to Equation (1) can also be found in the literature [55,56,57,58,59,60,61,62,63,64,65,66].

As per the requirements delineated in JSSG206 [11], MIL-STD-1530D [12], and USAF Structures Bulletin EZ-SB-19-01 [13], to follow a “building block approach”, the coefficients *D*, *A*, and *n* in Equation (1) were taken from prior studies [10,16]: *D* = 1.86 × 10^−9^, *n* = 2 and *A* = 111 MPa √m.

The crack growth analysis of all twenty-five cracks in these specimens was performed using Equation (1). As in previous papers [10,16], at each stage in the analysis, the stress intensity factor distribution around the crack tip was determined using the three-dimensional finite element alternating approach [67,68,69], and the change in the (three-dimensional) shape of the crack was computed using Equation (1). The advantage of using the three-dimensional finite element alternating method is that the cracks are not modelled explicitly and, regardless of the shape of the crack, only the uncracked finite element model is needed [67,69]. As such, this approach is ideal for assessing fatigue crack growth. However, since Equation (1) is now available in the commercial finite element programs ABAQUS^®^, NASTRAN^®^, and ANSYS^®^ via the Zencrack^®^ software module [70], an alternative approach could have been to use conventional finite element analyses in conjunction with the Zencrack^®^ software program. 

Consequently, in order to determine the stress intensity factors for any given crack configuration, it was first necessary to develop a three-dimensional finite element model of the repaired structure. In this analysis, the Young’s modulus and Poisson’s ratio of 7075-T7351 were taken to be 73,000 MPa and 0.3, respectively. The Young’s modulus and Poisson’s ratio of the cold spray deposit were taken from [16], and considered to be 69,000 MPa and 0.3, respectively. The maximum principal stress in the repaired specimen corresponding to a remote load of 30 kN, as determined using NASTRAN, is shown in Figure 3. 

## 3. Comparison of the Measured and Computed Crack Growth Histories

The failure surfaces associated with these twelve fatigue tests, as well as the comparisons between the measured and computed crack growth histories for each of the resultant twenty-five cracks, are shown in Figure 4, Figure 5, Figure 6, Figure 7, Figure 8, Figure 9, Figure 10, Figure 11, Figure 12, Figure 13, Figure 14, Figure 15, Figure 16, Figure 17, Figure 18, Figure 19, Figure 20, Figure 21, Figure 22, Figure 23, Figure 24, Figure 25, Figure 26, Figure 27, Figure 28, Figure 29, Figure 30, Figure 31, Figure 32, Figure 33, Figure 34, Figure 35, Figure 36, Figure 37, Figure 38 and Figure 39. The crack identifiers associated with each of the cracks shown in these figures are listed in Table 3, along with the starting crack sizes used in each of the analyses. 

### 3.1. Measured and Computed Results for the Specimens Used Spectrum 1

1. Specimen Number 75_1_NC_1_#1

**Figure 4:**
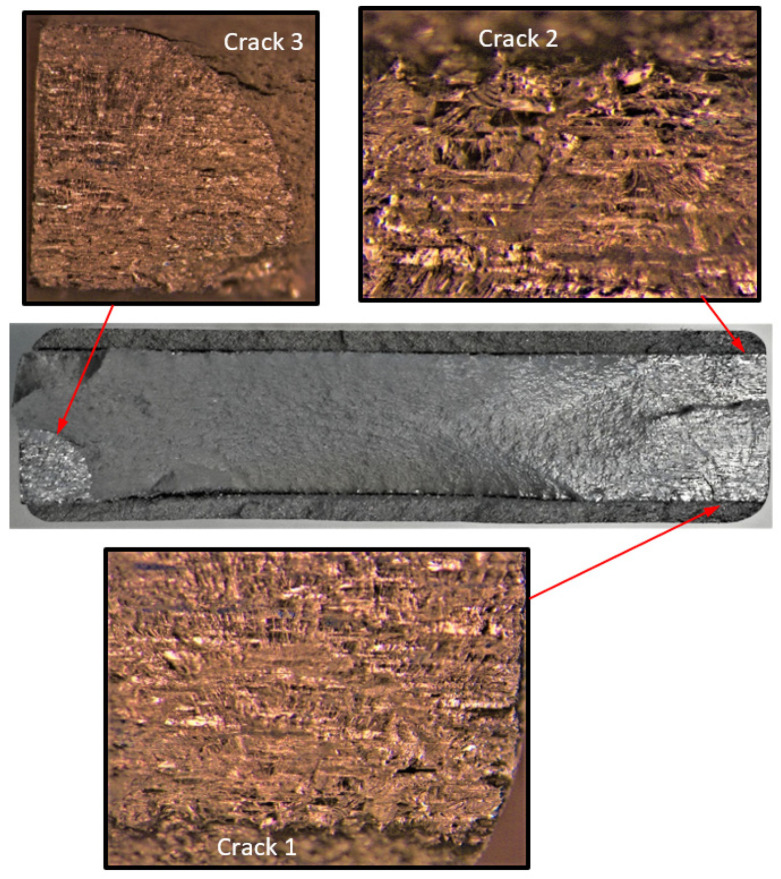
Failure in the 75_1_NC_1_#1 specimen (failed 368,528 cycles).

**Figure 5:**
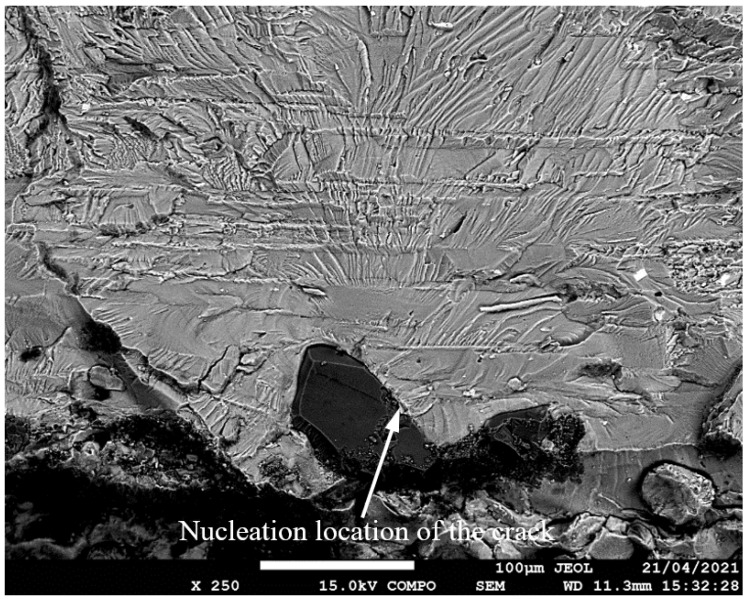
SEM of Crack 1, which was the fastest-growing (i.e., the lead) crack in specimen 75_1_NC_1_#1.

**Figure 6:**
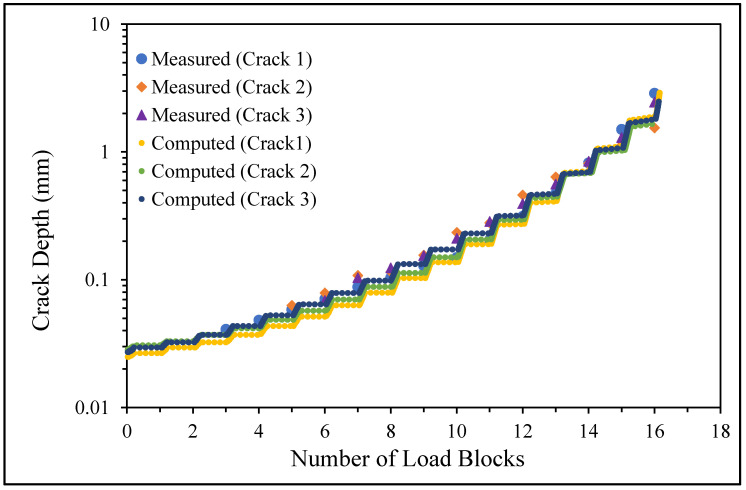
The measured and computed crack depth histories for specimen 75_1_NC_1_#1.

2. Specimen Number 75_1_NC_2_#2

**Figure 7:**
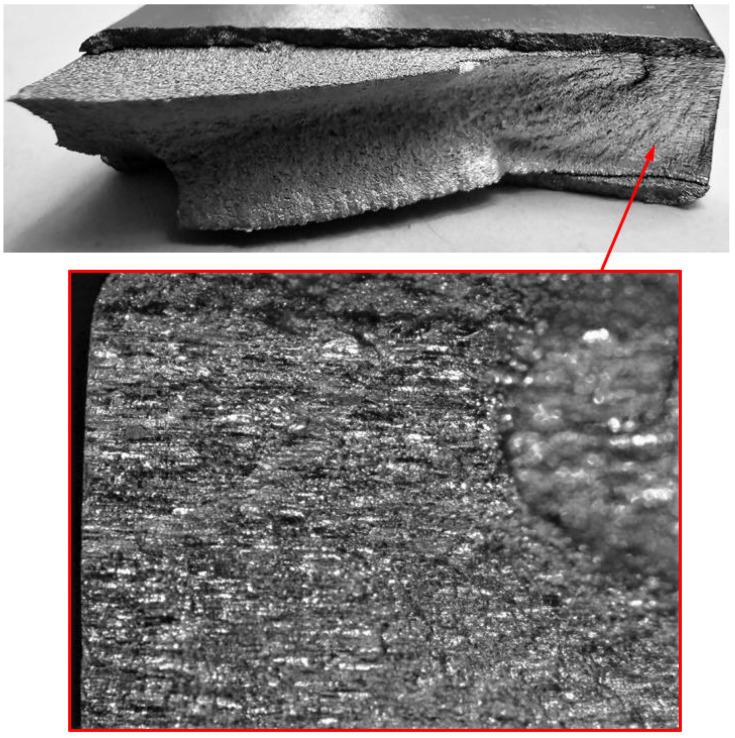
Failure in the 75_1_NC_2_#2 specimen (failed 354,393 cycles).

**Figure 8:**
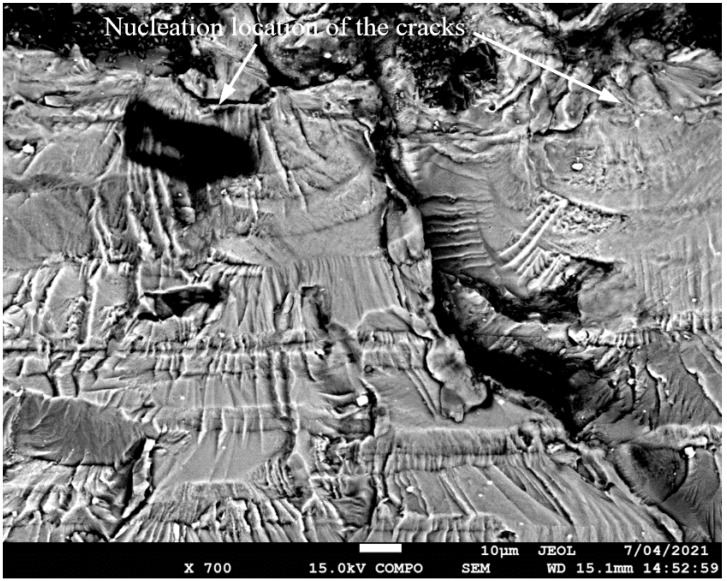
SEM of crack in specimen 75_1_NC_2_#2.

**Figure 9:**
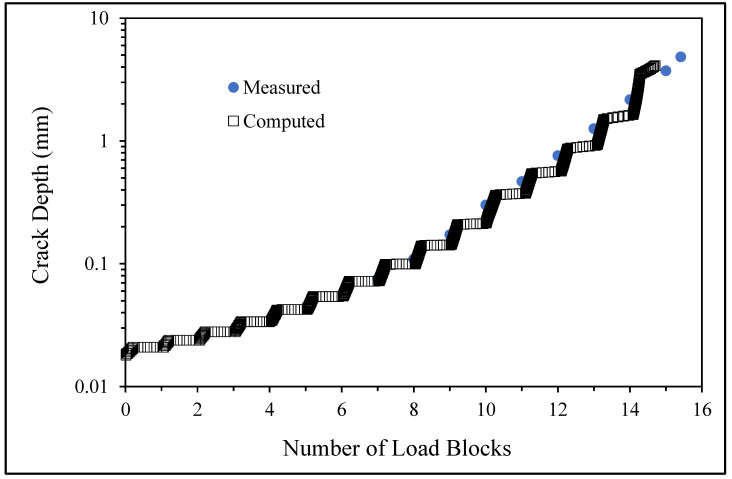
The measured and computed crack depth histories for specimen 75_1_NC_2_#2.

### 3.2. Measured and Computed Results for the Specimens Tested under Spectrum 2

1. Specimen Number 75_1_NC_1_#2

**Figure 10:**
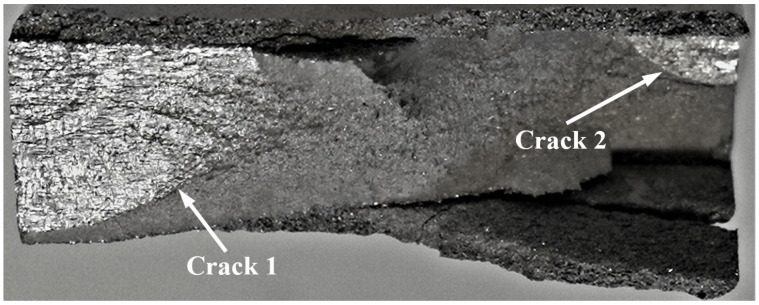
Failure in the 75_1_NC_1_#2 specimen (failed 378,932 cycles).

**Figure 11:**
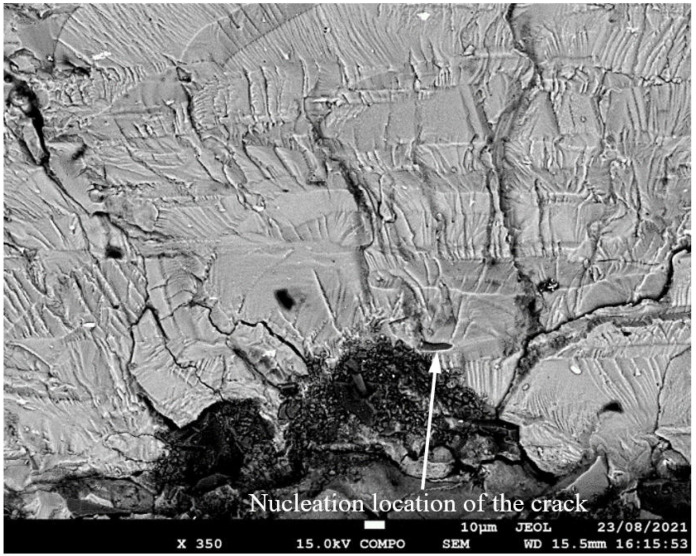
SEM of the lead crack (Crack 1) in specimen 75_1_NC_1_#2.

**Figure 12:**
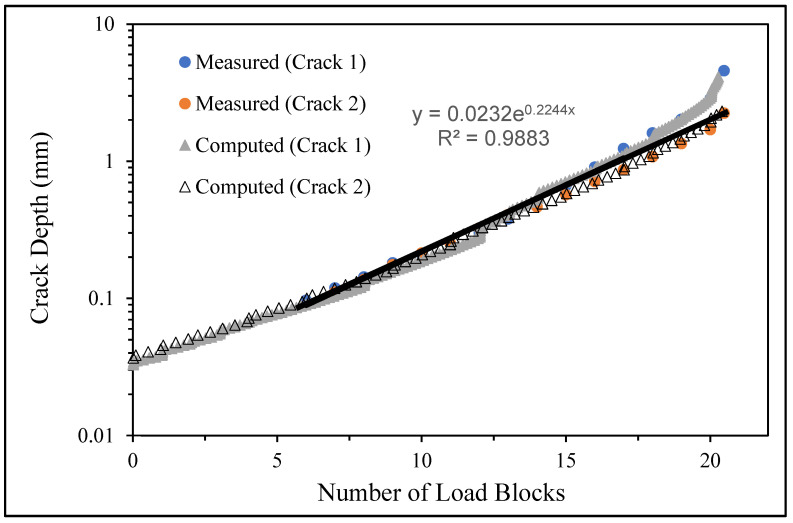
The measured and computed crack depth histories for specimen 75_1_NC_1_#2.

### 3.3. Measured and Computed Results for the Specimens Using Spectrum 3

1. Specimen Number 75_1_NC_2_#3

**Figure 13:**
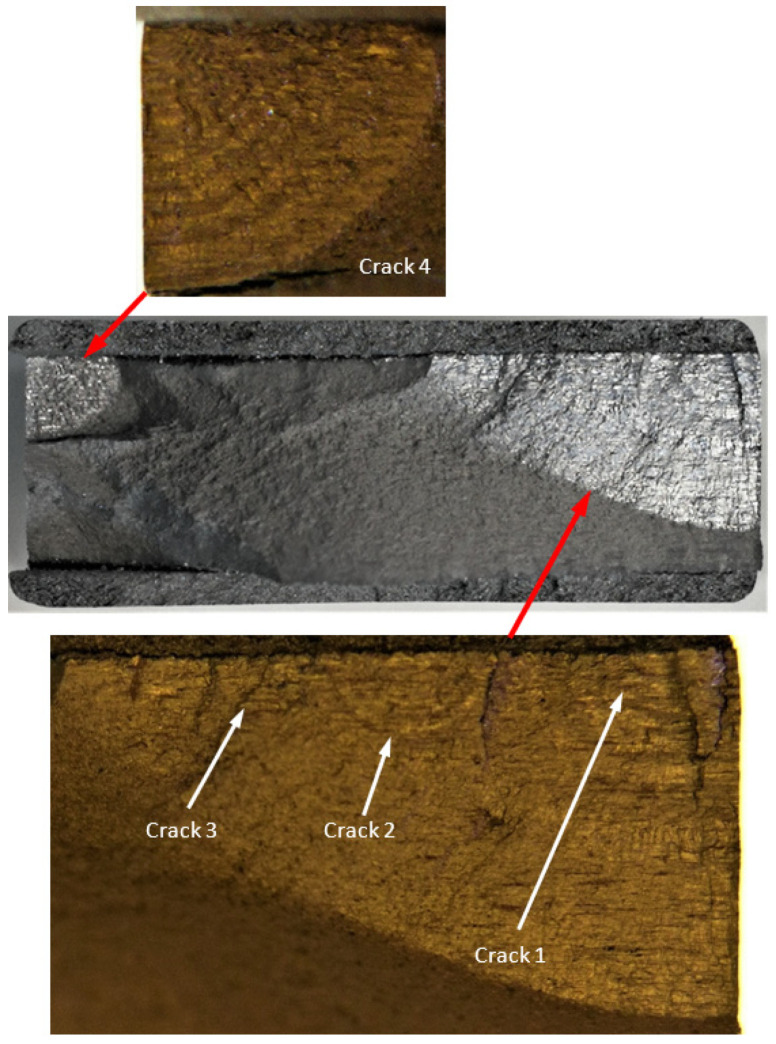
Failure in the 75_1_NC_2_#3 specimen (failed 365,534 cycles).

**Figure 14:**
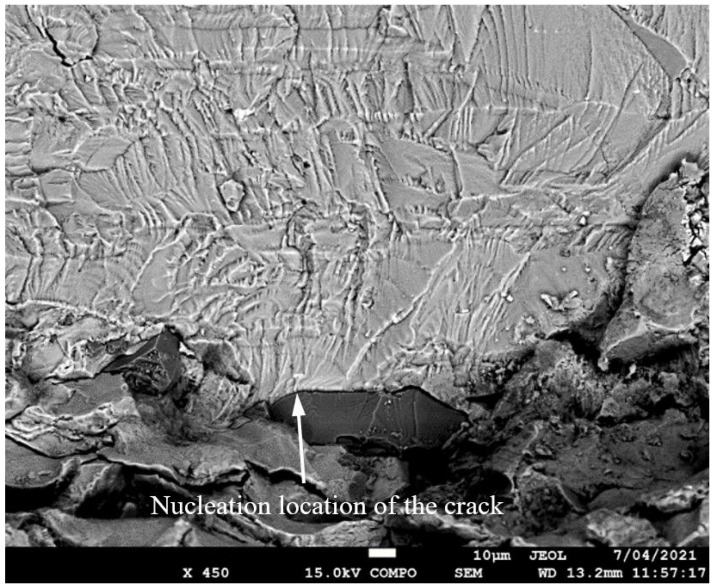
SEM of the lead crack (Crack 1) in specimen 75_1_NC_2_#3.

**Figure 15:**
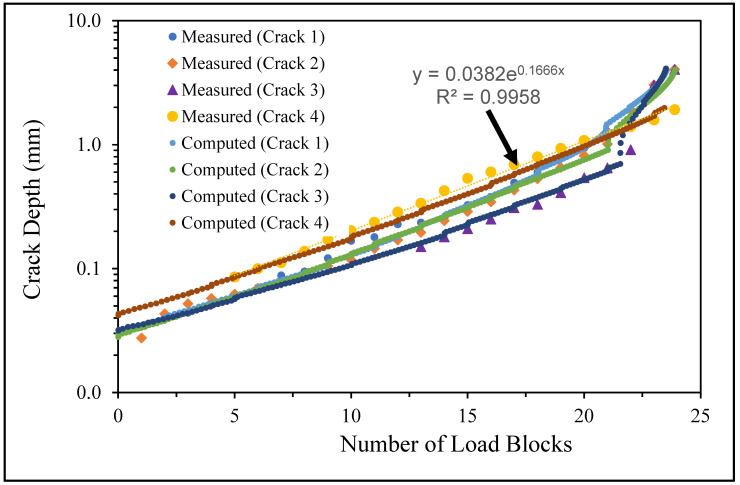
The measured and computed crack depth for specimen 75_1_NC_2_#3.

2. Specimen Number B_1_1_#1

**Figure 16:**
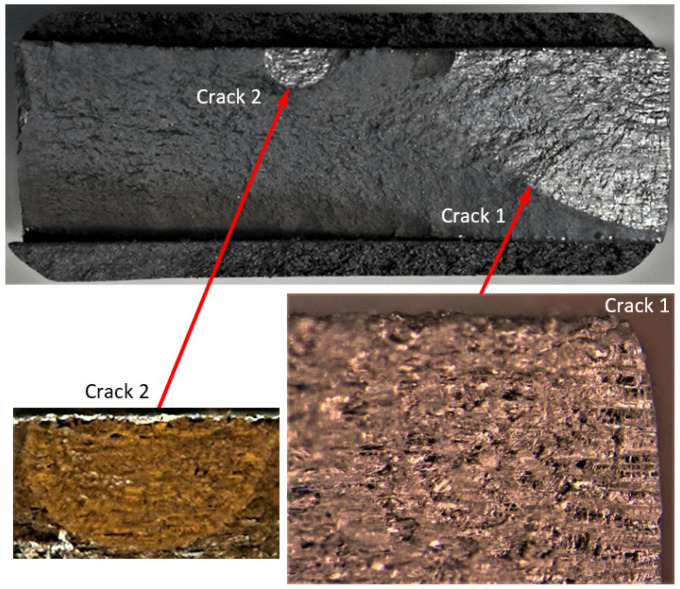
Failure in the B_1_1_#1 specimen (failed 555,619 cycles).

**Figure 17:**
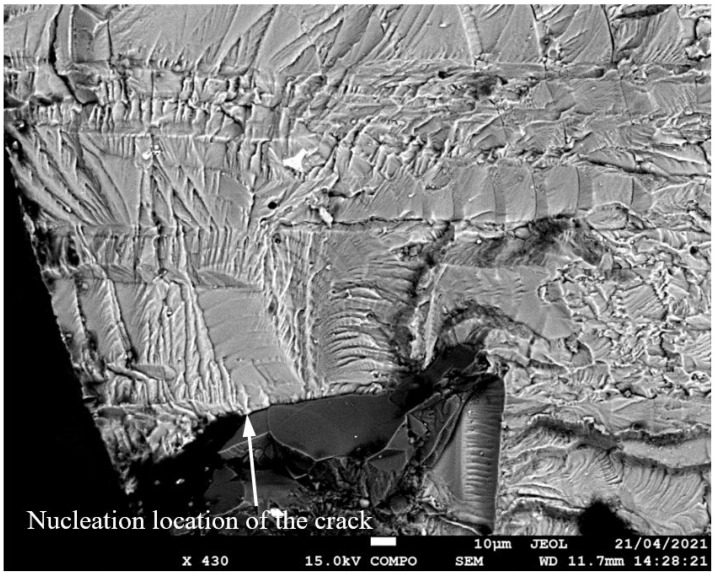
SEM of the lead crack (Crack 1) in specimen B_1_1_#1.

**Figure 18:**
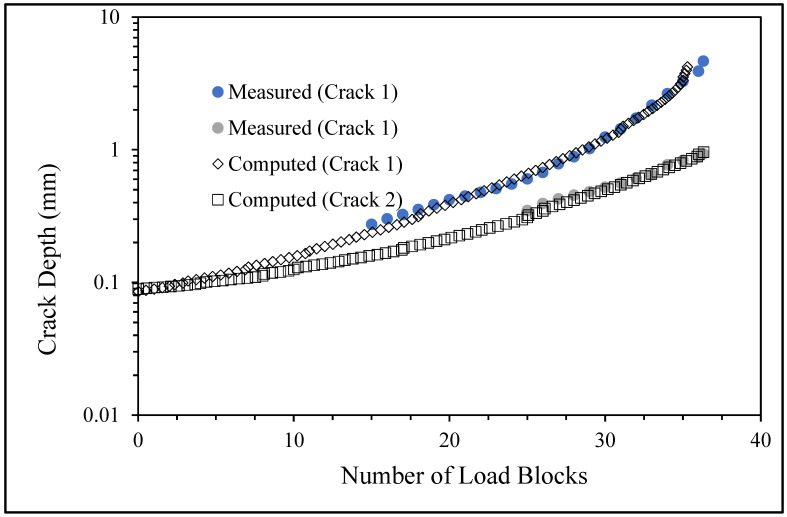
The measured and computed crack depth histories for specimen B_1_1_#1.

### 3.4. Measured and Computed Results for the Specimens Used Spectrum 4

1. Specimen Number 75_1_NC_1_#3

**Figure 19:**
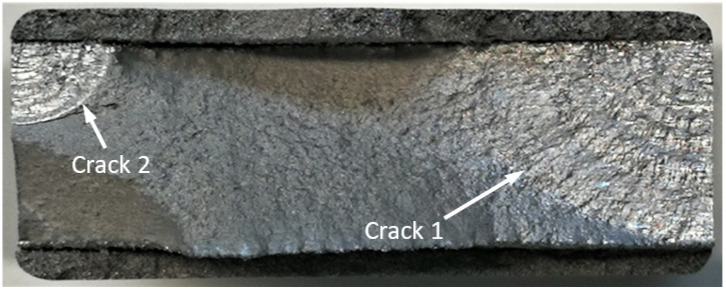
Failure in the 75_1_NC_1_#3 specimen (failed 629,120 cycles).

**Figure 20:**
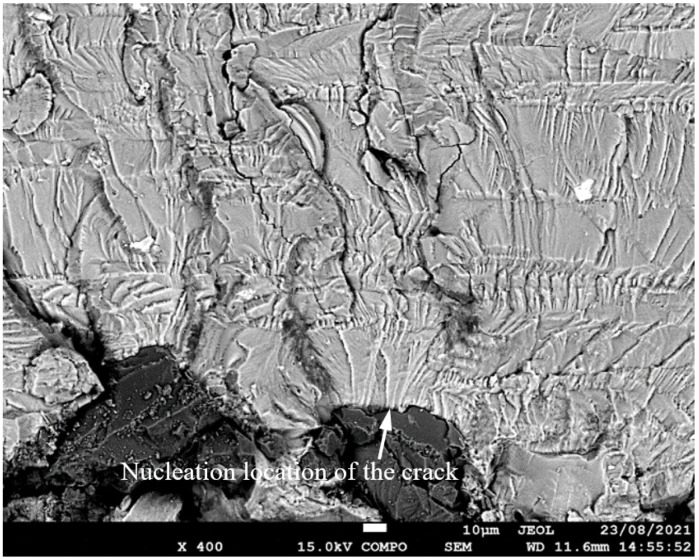
SEM of the lead crack (Crack 1) in specimen 75_1_NC_1_#3.

**Figure 21:**
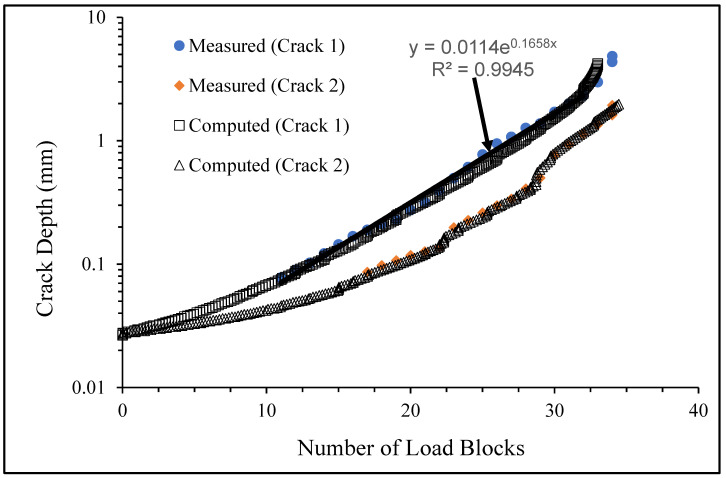
The measured and computed crack depth histories for specimen 75_1_NC_1_#3.

### 3.5. Measured and Computed Results for the Specimens Used Spectrum 5

1. Specimen Number 75_1_NC_1_#4

**Figure 22:**
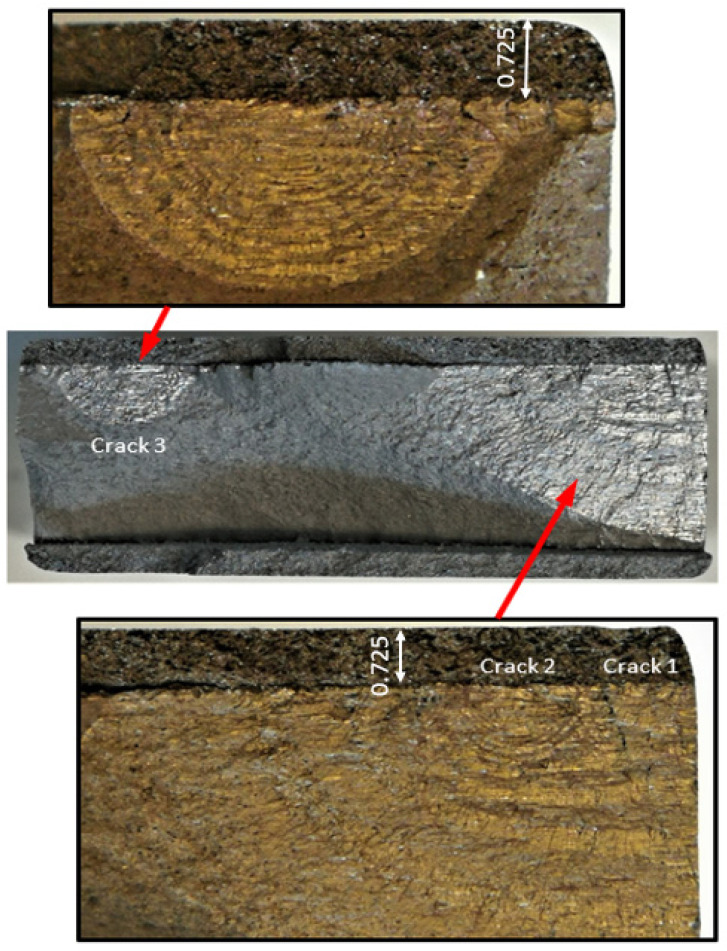
Failure in the 75_1_NC_1_#4 specimen (failed 670,905 cycles).

**Figure 23:**
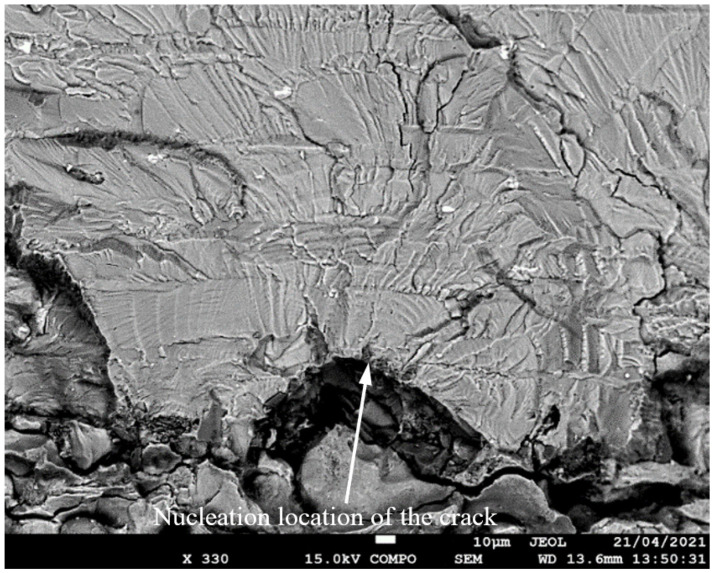
SEM of the lead crack (Crack 1) in specimen 75_1_NC_1_#4.

**Figure 24:**
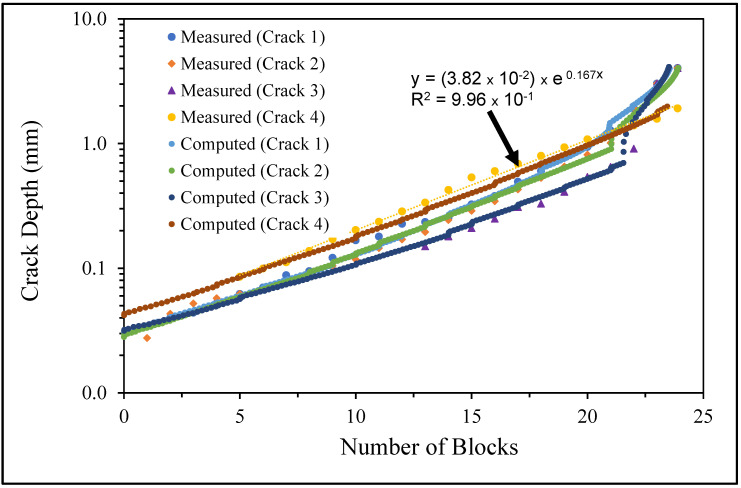
The measured and computed crack depth histories for specimen 75_1_NC_1_#4.

2. Specimen Number 75_1_NC_2_#4

**Figure 25:**
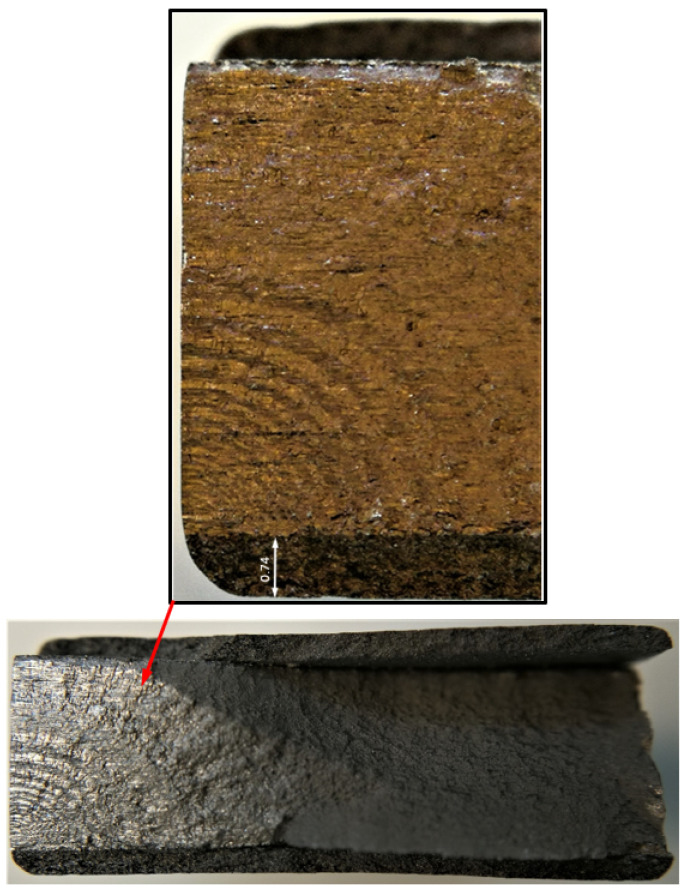
Failure in the 75_1_NC_2_#4 specimen (failed 360,543 cycles).

**Figure 26:**
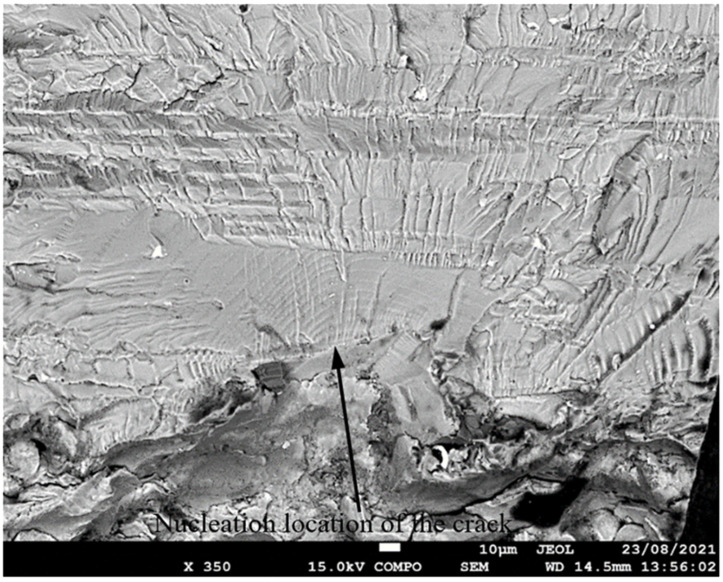
SEM of the lead crack in specimen 75_1_NC_2_#4.

**Figure 27:**
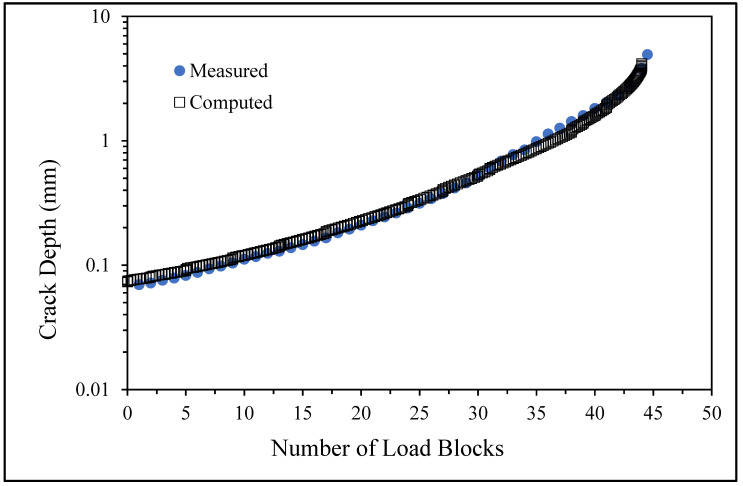
The measured and computed crack depth histories for specimen 75_1_NC_2_#4.

3. Specimen Number 75_1_NC_2_#5

**Figure 28:**
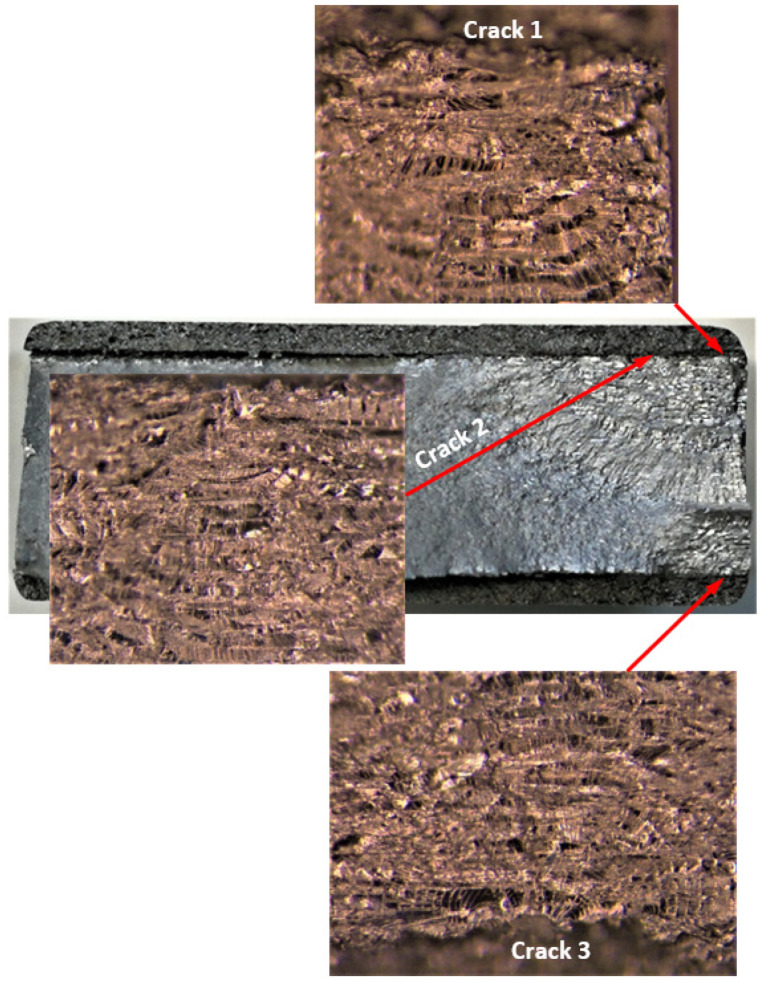
Failure in the 75_1_NC_2_#5 specimen (failed 373,397 cycles).

**Figure 29:**
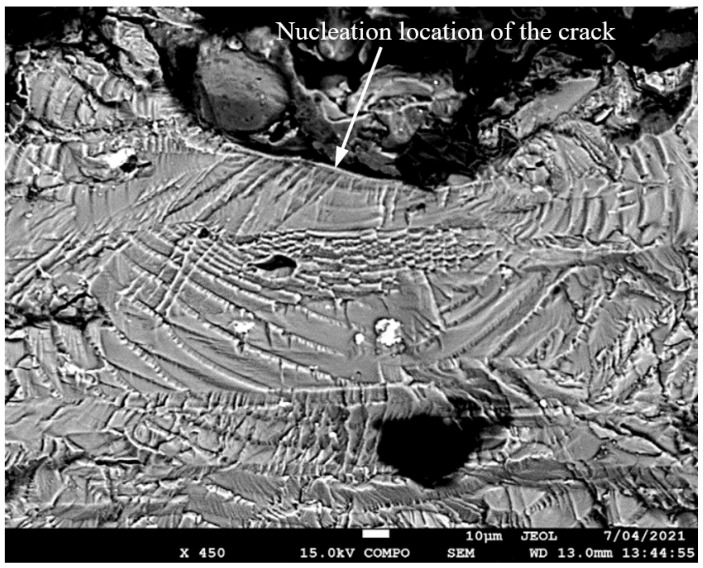
SEM of the lead crack (Crack 1) in specimen 75_1_NC_2_#5.

**Figure 30:**
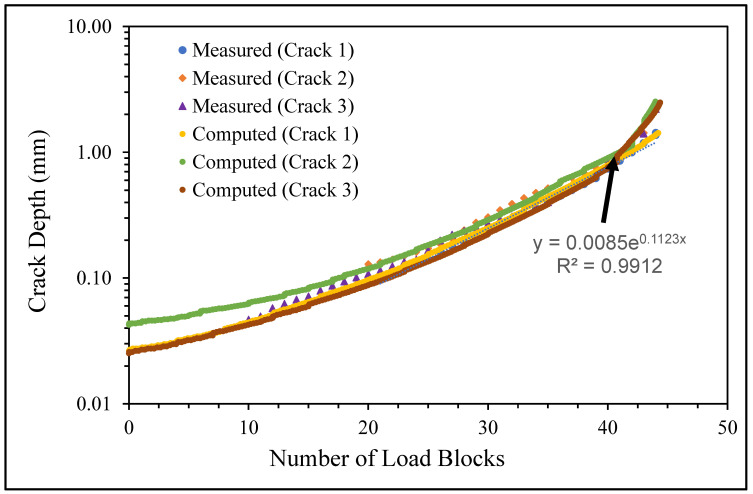
The measured and computed crack depth histories for specimen 75_1_NC_2_#5.

4. Specimen Number B_1_1_#2

**Figure 31:**
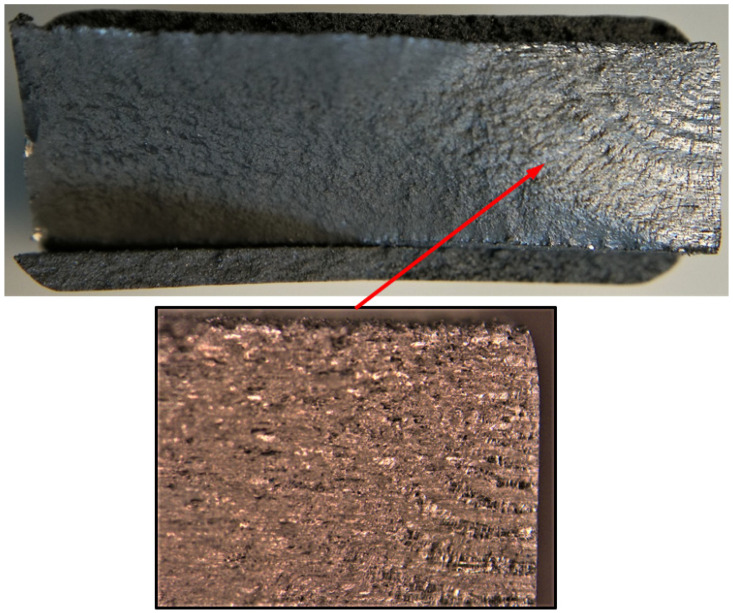
Failure in the B_1_1_#2 specimen (failed 1,117,056 cycles).

**Figure 32:**
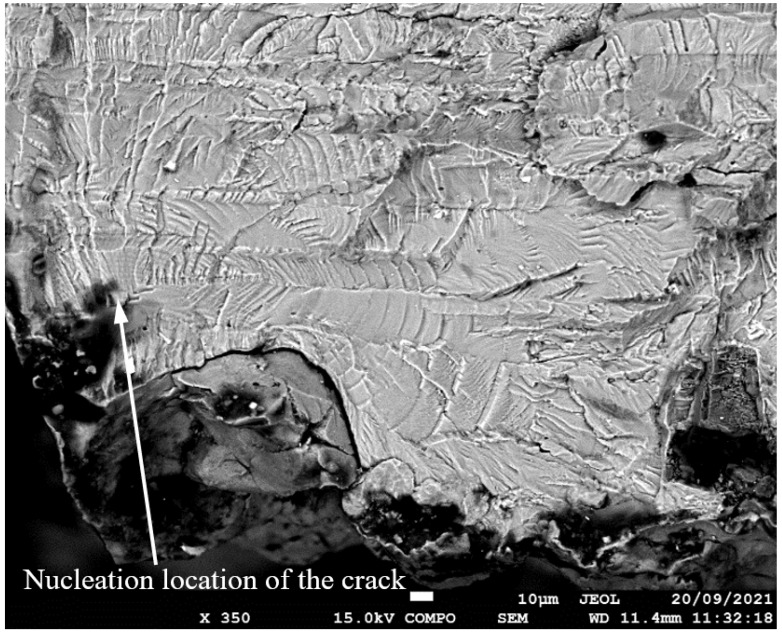
SEM of the lead crack in specimen B_1_1_#2.

**Figure 33:**
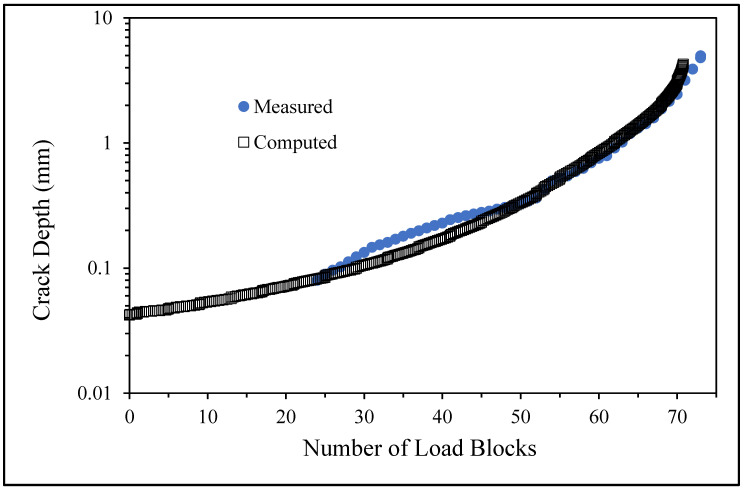
The measured and computed crack depth histories for specimen B_1_1_#2.

5. Specimen Number B_1_1_#3

**Figure 34:**
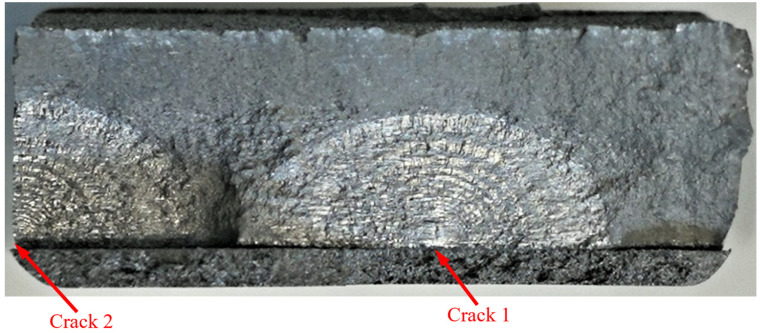
Failure in the B_1_1_#3 specimen (failed 765,288 cycles).

**Figure 35:**
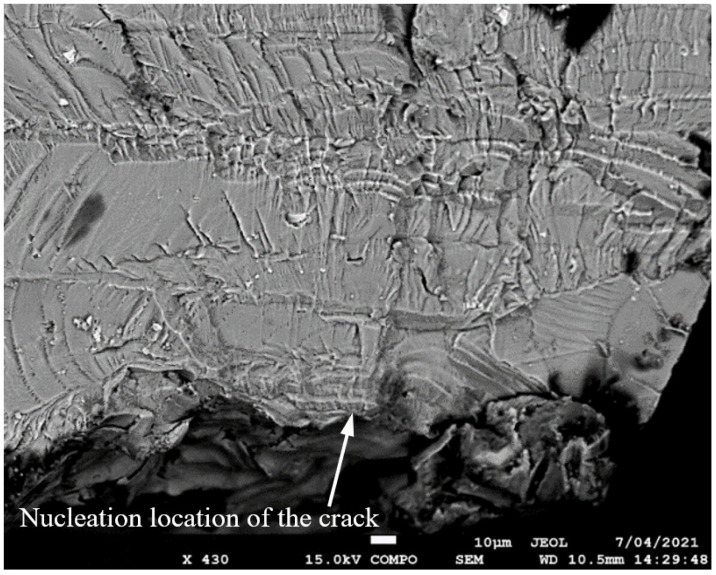
SEM of the lead crack (Crack 2) in specimen B_1_1_#3.

**Figure 36:**
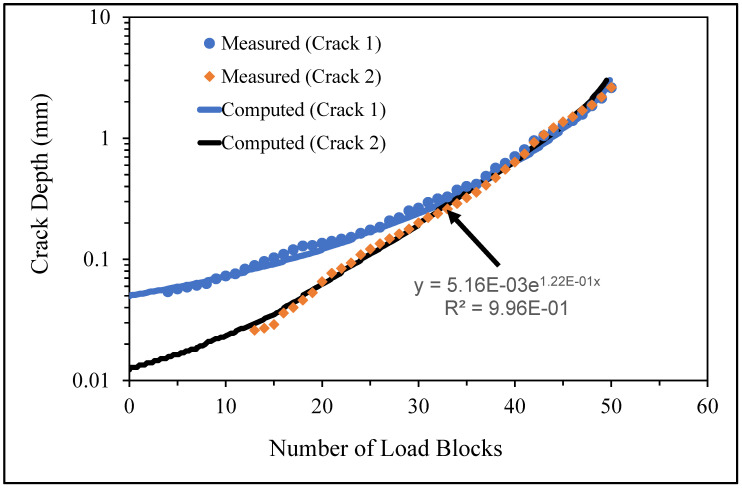
The measured and computed crack depth histories for specimen B_1_1_#3.

### 3.6. Measured and Computed Results for the Specimens Used Spectrum 6

1. Specimen Number 75_1_NC_1_#5

**Figure 37:**
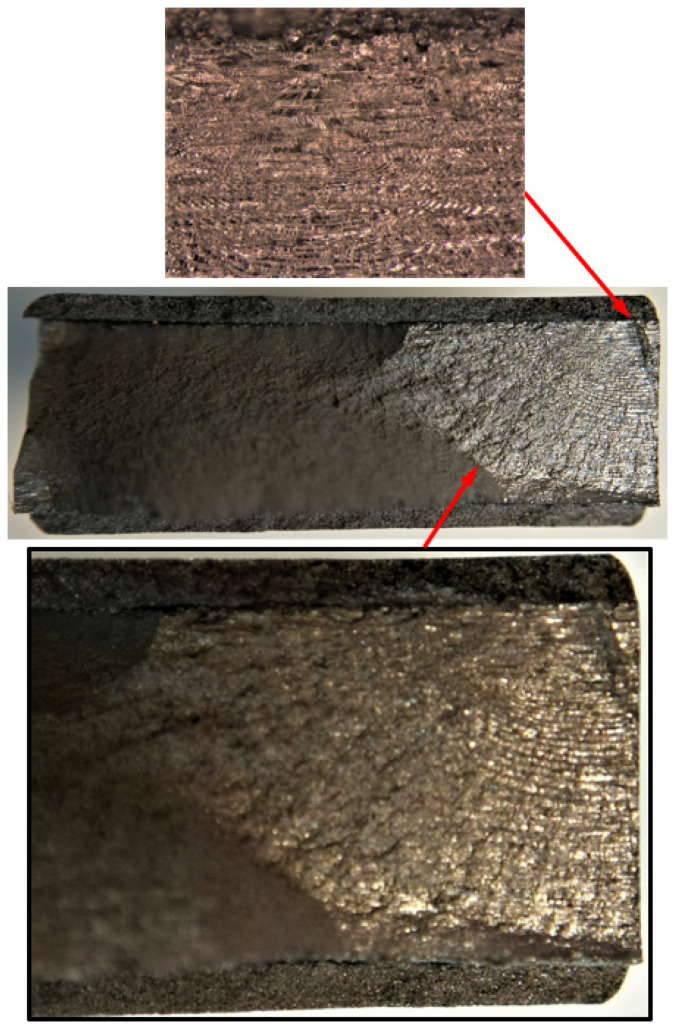
Failure in the 75_1_NC_1_#5 specimen (failed 899,480 cycles).

**Figure 38:**
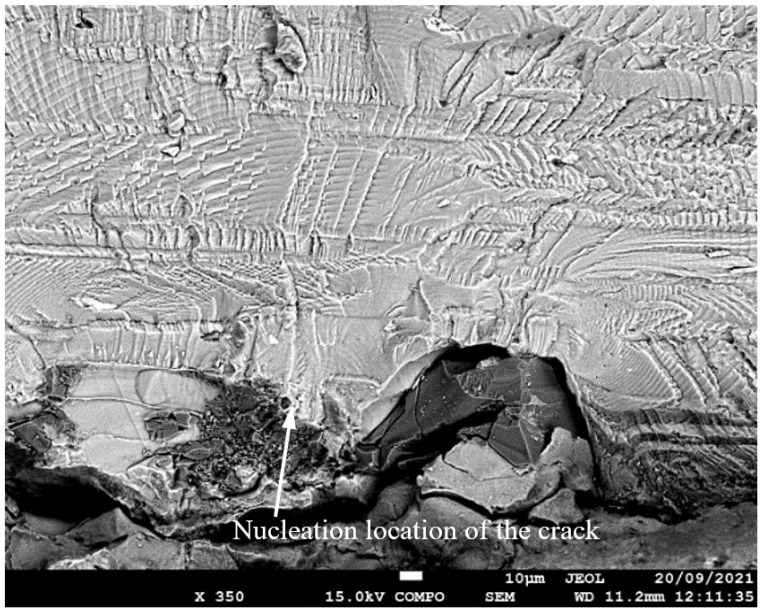
SEM of the lead crack in specimen 75_1_NC_1_#5.

**Figure 39:**
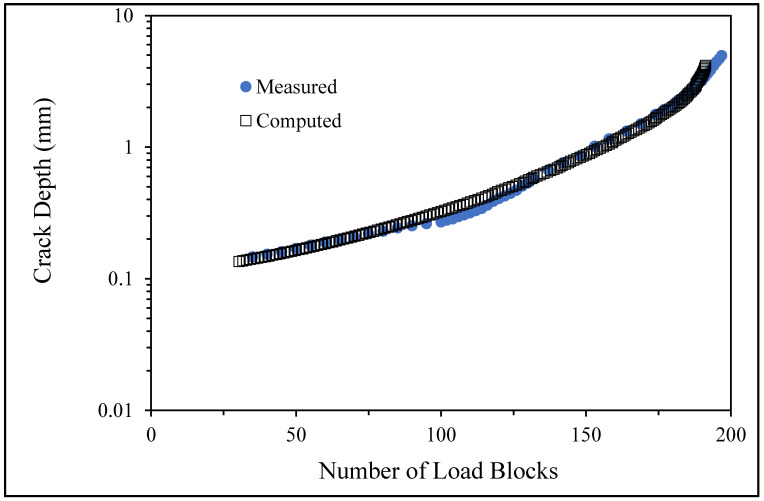
The measured and computed crack depth histories for specimen 75_1_NC_1_#5.

## 4. Assessment of the Computed versus Measured Crack Growth Histories 

There are a number of important conclusions that arise from this study, and these are as follows:

(i) The sizes of the nucleating cracks are significantly smaller than the mandated minimum equivalent initial damage size (EIDS) given in [11,12,13], namely 0.254 mm (0.01 inch). Hence, the analysis could be used to compute the growth of cracks from the mandated size, i.e., EIDS = 0.254 mm (0.01 inch).
(ii) The computed and measured crack growth histories agree well for each of the twenty-five cracks.
(iii) Table 3 reveals that the value of Δ*K_thr_* associated with the majority of the twenty-five cracks falls within the range of 0.0 to (approximately) 0.3 MPa √m, which is commonly seen for small cracks in conventionally manufactured aluminium alloys [21,23,25,64,71], as well as for small naturally occurring cracks in additively manufactured parts [37,72,73,74,75].
(iv) Figure 12, Figure 15, Figure 24, Figure 30 and Figure 36 reinforce the prior finding that the fastest-growing cracks, which are also called “lead cracks” [76,77], in cold spray repairs to simulated corrosion damage have a crack growth history that is approximately exponential.

The fracture mechanics explanation as to why small naturally occurring cracks that have a small value of Δ*K_thr_* often exhibit near exponential growth is given in a previous paper [21]. Examples of this phenomenon are also given [73,74,75] for the growth of small naturally occurring cracks in additively manufactured metals. This phenomenon can also be seen in the study by Gallagher et al. [78], which used the USAF Characteristic *K* approach, as delineated in the USAF Damage Tolerant Design handbook [79], together with the assumption that the fatigue threshold was zero to analyse the crack growth in 7075-T7451 specimens subjected to six different combat aircraft flight load spectra. Further examples of the ability of the Hartman–Schijve crack growth equation and the characteristic *K* approach, or variants thereof, to accurately represent the growth of small naturally occurring cracks in conventionally built metals and the resultant exponent nature of the crack growth history are discussed by Molent [80]. At this point, it should also be noted that exponential growth is consistent with the growth of lead cracks seen in both USAF and Royal Australian Air Force (RAAF) operational aircraft [77,81]. 

Figure 6 and Figure 9 also reveal that, for the spectra investigated, cracks that have relatively small Δ*K_thr_* values can yield a crack growth history that has a “staircase”-like shape, i.e., has changes in slope as the loads change within a given load block. 

### Variability in the Crack Growth Curves and NASA-HDBK-5010

As previously noted, the MIL-STD-1530D mandates that the airworthiness certification of an airframe must be based on linear elastic fracture mechanics, and USAF Structures Bulletin EZ-SB-19-01 states the same thing for additively manufactured parts and modifications. USAF Structures Bulletin EZ-SB-19-01, which addresses the airworthiness certification requirements for AM parts and, by implication, for cold spray repairs, highlights the importance of accounting for the variability in crack growth. In this context, it should also be noted that the study by Virkler, Hillberry and Goel [82] is acknowledged as being one of the first to highlight the extent of the variability in the *da*/*dN* versus Δ*K* curves associated with ASTM E647 [83] fatigue tests on long cracks in conventionally manufactured metals. On the other hand, previous work in [84] was the first to show the extent of this variability for cracks that emanated from etch pits that had an EIDS similar to that mandated in MIL-STD-1530D and USA Structures Bulletin EZ-SB-19-01. As a result of the acknowledged variability in the growth of cracks in conventionally manufactured materials, NASA Fracture Control Handbook NASA-HDBK-5010 [18] mandates that the *da*/*dN* versus Δ*K* curve used in any crack growth assessment must be the worst-case curve. 

**Figure 40:**
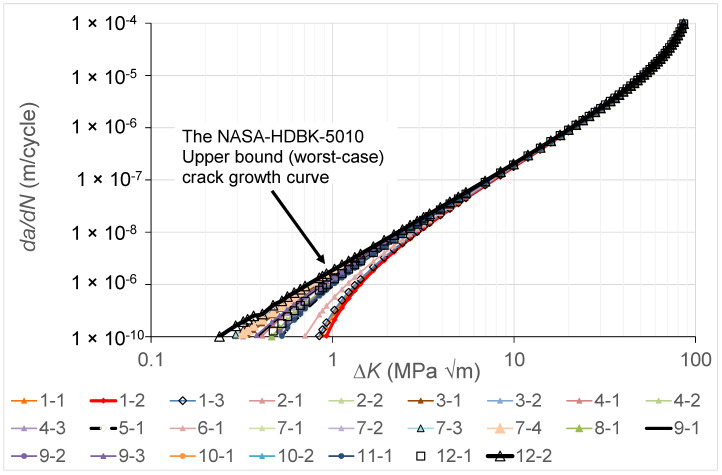
The variability in the crack growth curves seen by the twenty-five cracks examined in the present study.

The variability in the twenty-five *da*/*dN* versus Δ*K* curves determined in the current study is shown in Figure 40. This enables us to determine the NASA-HDBK-5101 mandated worst-case *da*/*dN* versus Δ*K* curve. It should be noted that this is the first time that the extent of the variability in the *da*/*dN* versus Δ*K* curves associated with cold spray repairs, where the nucleating crack lay at the intersection between the cold spray deposit and the substrate, has been shown. This worst-case curve is also shown in Figure 40. 

The (relatively large) variation in the crack growth curves associated with low values of *da*/*dN* highlights the fact that, before any durability assessment of a cold spray repair to an operational airframe is attempted, it is first necessary to perform a sufficient number of tests so that the worst-case crack growth curve needed to perform the mandated airworthiness certification assessment can be determined.

## 5. Conclusions

MIL-STD-1530D notes that analysis is the key to certification, and that the role of testing is merely to validate and assist in correcting the analysis. USAF Structures Bulletin EZ-SB-19-01, MIL-STD-1530D and the US Joint Services Structural Guidelines JSSG2006 mandate that a durability analysis must result in an EIDS of no greater than 0.254 mm (0.01 inch). Furthermore, the durability analysis must be consistent with the building block approach outlined in MIL-STD-1530D and the US Joint Services Structural Guidelines JSSG2006. 

This study has confirmed the ability to accurately compute the crack growth histories, i.e., to accurately perform the durability analyses mandated in JSSG2006, MIL-STD-1530Dc, and USAF Structures Bulletin EZ-SB-19-01, associated with twenty-five naturally occurring cracks that had nucleated and subsequently grown from the material discontinuities associated with cold spray repairs to simulated corrosion damage. Furthermore, in these tests, the size of the nucleating cracks was either comparable to, or smaller than, the equivalent initial damage size (EIDS) mandated in the JSSG2006, MIL-STD-1530D, and USAF Structures Bulletin EZ-SB-19-01. It should also be noted that the durability analysis described in this study has followed the building block approach outlined in MIL-STD-1530D and in the US Joint Services Structural Guidelines JSSG2006, and that the variability in the crack growth histories is captured by allowing for variability in the local fatigue threshold.

We also illustrate the extent of the variability in the *da*/*dN* versus Δ*K* curves associated with cold spray repairs; to the best of the authors’ knowledge, this is the first time that this variability has been reported. The relatively large variation in the *da*/*dN* versus Δ*K* curves associated with low values of *da*/*dN* highlights the fact that, before any durability assessment of a cold spray repair to an operational airframe is attempted, it is first necessary to perform a sufficient number of tests so that the worst-case small crack growth curve needed to perform the mandated airworthiness certification analysis can be determined.

## Figures and Tables

**Figure 1:**
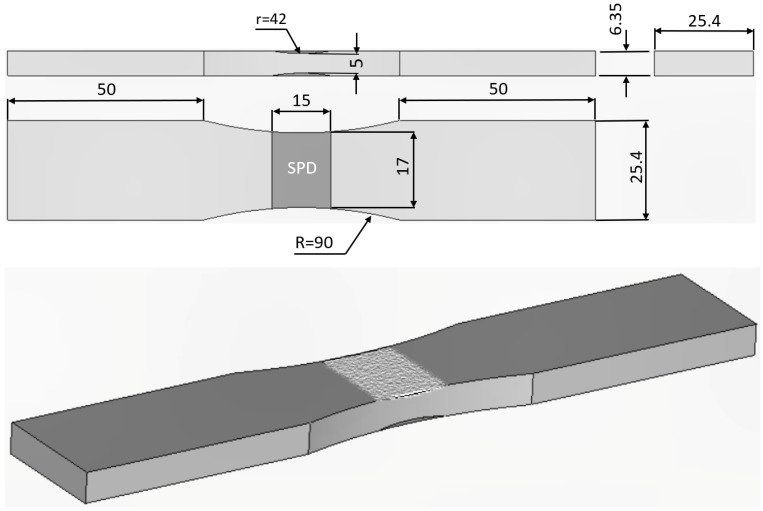
Dimensions of the test specimen geometry.

**Figure 2:**
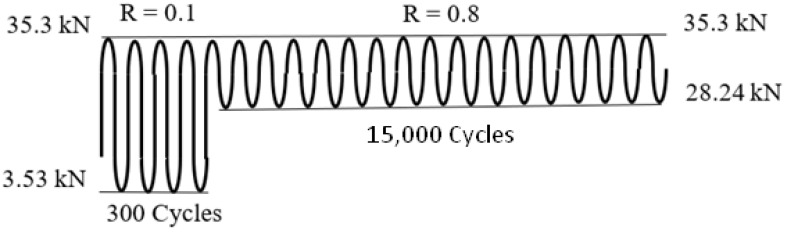
A schematic diagram of marker block load spectrum 3.

**Figure 3:**
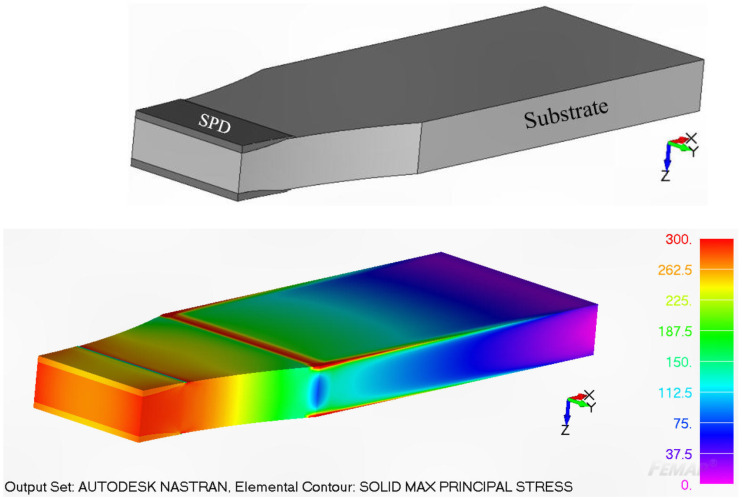
The maximum principal stress in the specimen at a remote load of 30 kN. Only one half of the specimen is shown.

**Table 2:** The block loading spectra used in the various crack growth tests and the thicknesses of the cold spray.

Specimen ID	Block Loading Spectrum	Thickness of Cold Spray Deposit (+/−0.005 mm)
75_1_NC_1_#1	1	0.785
75_1_NC_1_#2	2	0.755
75_1_NC_1_#3	4	0.76
75_1_NC_1_#4	5	0.73
75_1_NC_1_#5	6	0.72
75_1_NC_2_#2	1	0.818
75_1_NC_2_#3	3	0.735
75_1_NC_2_#4	5	0.74
75_1_NC_2_#5	5	0.73
B_1_1_#1	3	0.935
B_1_1_#2	5	0.925
B_1_1_#3	5	0.14

**Table 3:** The starting crack length and the values of Δ*K_thr_* (MPa √m) used in the crack growth predictions.

Specimen ID	Crack ID	Simplified Specimen and Crack Identifier	Starting Crack Length (mm)	Δ*K_thr_* (MPa √m)
75_1_NC_1_#1	1	1-1	0.0246	0.64
	2	1-2	0.0280	0.69
	3	1-3	0.0269	0.61
75_1_NC_1_#2	1	2-1	0.0327	0.09
	2	2-2	0.0353	0.10
75_1_NC_1_#3	1	3-1	0.0260	0.10
	2	3-2	0.0261	0.173
75_1_NC_1_#4	1	4-1	0.0180	0.135
	2	4-2	0.0330	0.176
	3	4-3	0.0780	0.33
75_1_NC_1_#5	1	5-1	0.0463	0.29
75_1_NC_2_#2	1	6-1	0.0180	0.47
75_1_NC_2_#3	1	7-1	0.0300	0.06
	2	7-2	0.0275	0.07
	3	7-3	0.0310	0.05
	4	7-4	0.0410	0.07
75_1_NC_2_#4	1	8-1	0.0726	0.23
75_1_NC_2_#5	1	9-1	0.0263	0.150
	2	9-2	0.0342	0.252
	3	9-3	0.0255	0.143
B_1_1_#1	1	10-1	0.0800	0.268
	2	10-2	0.0839	0.32
B_1_1_#2	1	11-1	0.0400	0.275
B_1_1_#3	1	12-1	0.0480	0.20
	2	12-2	0.0120	0.05

## Data Availability

The data will be made available at the completion of the project.
